# Virtual reality for reducing intraoperative anxiety and improving patient satisfaction under regional anesthesia among palestinian patients: a randomized controlled trial

**DOI:** 10.1186/s12871-025-03475-3

**Published:** 2025-11-27

**Authors:** Khulud Mansor, Sayeda Ahmed Abdellatif, Nareman Aly Mohamed

**Affiliations:** 1 Department of Nursing, Ministry of Health, Nablus, Palestine; 2 Faculty of Nursing, Cairo University, Cairo, Egypt

**Keywords:** Virtual Reality, Anxiety, Patient Satisfaction, Regional Anesthesia, Palestine, Stress Reduction

## Abstract

**Background:**

Surgery and anesthesia are associated with heightened anxiety, particularly in procedures performed under regional anesthesia where patients remain conscious. Perioperative anxiety can negatively affect hemodynamic stability, patient satisfaction, and overall recovery. Immersive virtual reality (VR) has emerged as a promising non-pharmacological intervention to manage intraoperative anxiety.

**Objectives:**

This study aimed to examine the effect of VR on intraoperative anxiety (primary endpoint), stress levels, and patient satisfaction (secondary endpoints) in Palestinian patients undergoing urological surgery under regional anesthesia.

**Methods:**

A total of 145 patients undergoing elective urological surgery under spinal or epidural anesthesia at Rafidia Governmental Hospital, Palestine, were enrolled in this assessor-blinded randomized controlled trial between June and September 2024. Participants were randomly assigned to a VR group (*n* = 72) or control group (*n* = 73). The primary endpoint was intraoperative anxiety measured using the State-Trait Anxiety Inventory (STAI) at 30 min post-induction. Secondary endpoints included stress (Perceived Stress Scale), patient satisfaction (Visual Analog Scale), and hemodynamic parameters.

**Results:**

The VR group demonstrated significantly lower intraoperative anxiety scores compared to the control group (37.2 ± 15.3 vs. 52.9 ± 15.7, *P* < 0.001, Cohen's d = 1.01). Secondary outcomes after Bonferroni correction (α = 0.0125) showed significantly lower stress levels (21.1 ± 5.3 vs. 25.9 ± 5.8, *P* < 0.001) and higher satisfaction scores (82.4 ± 12.1 vs. 61.3 ± 15.7, *P* < 0.001). Heart rate differences were not statistically significant after correction (85.5 ± 7.6 vs. 90.0 ± 7.8 bpm, *P* = 0.028).

**Conclusions:**

VR is a safe and effective non-pharmacological intervention that reduces intraoperative anxiety and stress while improving patient satisfaction in urological surgeries performed under regional anesthesia. These findings support VR integration into perioperative care protocols for procedures involving regional anesthesia.

**Trial Registration:**

Retrospectively registered at ClinicalTrials.gov with identifier NCT07132216 on August 20, 2025.

**Supplementary Information:**

The online version contains supplementary material available at 10.1186/s12871-025-03475-3.

## Introduction

Surgery and anesthesia are significant sources of stress and anxiety for patients, with perioperative anxiety being a prevalent psychological response [1]. Regional anesthesia, while advantageous for postoperative recovery and pain management, often leaves patients conscious and aware of their surroundings, exacerbating anxiety due to auditory and visual stimuli in the operating room [2, 3]. Studies report that 48–55% of surgical patients experience preoperative anxiety, with heightened levels linked to prolonged recovery, increased pain perception, and dissatisfaction with care [4, 5].

The psychological and physiological effects of anxiety during regional anesthesia are well-documented. Patients often fear loss of control, surgical complications, and pain, which can trigger stress responses such as elevated blood pressure, tachycardia, and increased cortisol levels [6, 7]. These responses may compromise surgical outcomes and delay recovery [8]. Traditional pharmacological interventions, such as benzodiazepines, carry risks of sedation, respiratory depression, and delayed postoperative recovery [9]. Non-pharmacological approaches are increasingly recognized as valuable alternatives for anxiety management [10].

Virtual reality (VR), an immersive technology that creates calming environments, has shown promise in reducing anxiety across clinical settings [11, 12]. Recent studies demonstrate VR's efficacy in reducing perioperative anxiety and improving satisfaction in procedures under regional anesthesia, such as cesarean sections and orthopedic surgeries [13, 14]. However, there is a notable lack of intraoperative VR studies in low-resource settings, particularly in Palestine, where cultural and contextual factors may influence VR's acceptability and effectiveness.

The theoretical framework of this study is grounded in Roy's Adaptation Model [15], which posits that individuals adapt to stressors through coping mechanisms. We hypothesized that VR would significantly reduce intraoperative anxiety compared to standard care, with concurrent improvements in stress levels and patient satisfaction, while maintaining hemodynamic stability.

## Methods

### Study design

This randomized controlled trial was conducted at Rafidia Governmental Surgical Hospital in Nablus, Palestine, from June to September 2024. The trial was retrospectively registered at ClinicalTrials.gov (NCT07132216, August 20, 2025). The protocol is available at https://clinicaltrials.gov/study/NCT07132216. The study followed a parallel-group design with 1:1 allocation ratio, testing VR intervention for superiority compared to standard care.

### Retrospective registration justification

The delay in trial registration occurred due to administrative challenges in accessing international registration platforms from Palestine and initial unfamiliarity with registration requirements. While this represents a limitation affecting transparency, all primary and secondary outcomes were pre-specified in the institutional protocol before patient recruitment. No post-hoc analyses were performed, and outcome definitions remained unchanged throughout the study period.

### Participants

#### Inclusion criteria

1. Adult patients (≥ 18 years) undergoing elective urological surgery under spinal or epidural anesthesia
2. No prior surgical history
3. Moderate to severe anxiety (STAI score > 38)
4. No cognitive impairments, able to understand and complete questionnaires
5. No contraindications to VR use

#### Exclusion criteria

1. History of psychiatric disorders, epilepsy, or chronic pain conditions
2. Severe cardiovascular/respiratory disease (ASA class ≥ III)
3. Use of anxiolytics, sedatives, or hypnotics before/during surgery
4. Intraoperative complications requiring conversion to general anesthesia
5. Technical failure of VR equipment during surgery

### Randomization and blinding

Block randomization with block size of 8 ensured balanced allocation using computer-generated sequences prepared by a statistician not involved in patient care. Sealed opaque envelopes were prepared and opened upon patient arrival. Participant enrollment was performed by research nurses, while allocation was concealed until assignment; investigators enrolling participants did not have access to the randomization sequence.

Patients were not blinded due to the nature of VR intervention, but anesthesiologists and outcome assessors remained blinded to group assignments. VR headsets were covered with opaque drapes during clinical assessments, and non-clinical staff managed VR equipment to maintain blinding.

### Ethical approval

The study was approved by the Ethics Committee of Scientific Research, Faculty of Nursing, Cairo University (IRB RHDIRB2019041701, November 27, 2024) and the Palestinian Ministry of Health (MOH-162/1512/2024, July 17, 2024). Written informed consent was obtained from all participants, with forms available in Arabic.

### Anesthesia protocol

The reported bupivacaine doses (15–20 mg) represent standard institutional practice for urological procedures at our center, comparable to doses used in similar regional centers. These doses are within established safety guidelines and were not protocol-specific modifications.

Standard monitoring included ECG, non-invasive blood pressure, SpO₂, and respiratory rate. Spinal anesthesia was administered using heavy bupivacaine 15–20 mg with fentanyl 12.5–25 mcg at L3-L4 or L4-L5 level. No sedatives were routinely administered to either group, though rescue sedation was available if clinically indicated.

### VR Intervention

The Meta Quest 2 VR headset was used with culturally adapted content. Potential Confounding of Spiritual Content: The VR intervention included Islamic prayer elements alongside nature scenes. We acknowledge this represents a potential confounding factor, as the observed effects may result from spiritual content rather than VR technology alone. An audio recording of Islamic prayer without VR might have produced similar effects. This limitation should be considered when interpreting results and represents an area for future research comparing VR with and without spiritual elements.

Patients selected from five nature environments (beach, forest, mountain, underwater, meadow) with 30-min guided meditation tracks in Arabic, incorporating culturally appropriate Islamic meditation techniques. Content was validated by local religious and cultural advisors.

VR was applied immediately after spinal anesthesia, before surgical incision, for 30 min with breaks if discomfort occurred. Patient tolerance to the VR headset was high, with minimal reports of simulator sickness as assessed by methods aligned with standard questionnaires [16].

### Control group

The control group received standard perioperative care without VR or additional interventions. Sedative Use Clarification: No patients in the control group received routine sedatives. Rescue sedation was available for both groups if clinically indicated, but none was required during the study period.

### Sample size calculation

Based on G*Power 3.1.9.7 with alpha 0.05, power 0.80, and effect size 0.5 from prior STAI studies [17], the minimum required sample was 64 per group (128 total). We enrolled 150 patients accounting for 10% attrition.

### Interim analyses

No interim analyses or stopping guidelines were planned or implemented.

### Outcomes

#### Primary endpoint

Intraoperative anxiety measured by STAI at 30 min post-anesthesia induction.

#### Secondary endpoints

- Stress levels (Perceived Stress Scale-10)
- Patient satisfaction (Visual Analog Scale 0–100 mm)
- Hemodynamic parameters (blood pressure, heart rate, respiratory rate, SpO₂)
- Adverse events

#### Patient satisfaction measurement limitation

A single-item VAS was used instead of comprehensive multi-dimensional tools (e.g., Patient Satisfaction Questionnaire-18) due to resource constraints. While this limits the depth of satisfaction assessment, single-item VAS scales demonstrate acceptable validity for overall satisfaction measurement in surgical settings and remain widely used in clinical research.

#### Adverse event assessment

Intraoperative monitoring findings and adverse events were systematically recorded in anesthesia charts, while postoperative events were documented in patient observation sheets during post-anesthesia care. All events were classified as either anesthesia-related or VR-related based on clinical assessment and timing. This systematic documentation aligns with rigorous reporting standards for clinical trials [18].

### Statistical analysis

Analysis was performed using SPSS version 27.

#### Baseline characteristics

Following recommendations for randomized trials, standardized differences rather than statistical tests were calculated for baseline characteristics to assess balance between groups.

#### Missing data

There were no missing data; all participants who completed follow-up were included in the analysis using intention-to-treat principles.

#### Statistical tests by endpoint

- Primary endpoint (STAI anxiety scores): Mann–Whitney U test for between-group comparisons, Wilcoxon signed-rank test for within-group changes
- Secondary endpoints: Independent t-tests for normally distributed continuous variables, Mann–Whitney U test for non-parametric data, Chi-square or Fisher's exact test for categorical variables

#### Effect sizes

Cohen's d was calculated for all primary and secondary outcomes. Confidence Intervals: 95% confidence intervals are reported for all effect sizes and primary outcomes.

#### Multiple comparisons

Given four secondary endpoints (stress, satisfaction, heart rate, and adverse events), Bonferroni correction was applied (adjusted alpha = 0.0125 for secondary outcomes). The primary endpoint maintained alpha = 0.05.

#### Additional analyses

No subgroup, sensitivity, or other additional analyses beyond prespecified outcomes were performed. All analyses were performed using IBM SPSS Statistics for Windows, Version 27.0 [19].

### Patient and public involvement

Although patients were not formally involved in trial design or conduct, patient-reported satisfaction outcomes were collected, and potential bias due to social desirability was acknowledged as a limitation.

### Protocol changes

No important protocol changes were made after trial commencement. All outcomes and analysis methods remained as originally specified.

### Trial completion

The trial was completed as planned without early termination.

### Reporting guidelines

This study adheres to CONSORT 2010 guidelines for reporting randomized controlled trials. A completed CONSORT checklist is provided as supplementary material.

## Results

### Participant flow

A total of 150 patients were assessed for eligibility, with 145 completing the study (VR group *n* = 72, control group *n* = 73). Five patients were excluded: three required conversion to general anesthesia and two experienced VR intolerance. No participants were lost to follow-up, resulting in complete data for all randomized participants who met inclusion criteria.

### Baseline characteristics

Baseline demographic and clinical characteristics showed good balance between groups (Table 1). Mean age was 36.1 ± 11.3 years (VR) versus 37.1 ± 12.5 years (control), with standardized difference of 0.08. Baseline anxiety (STAI: 52.8 ± 6.6 vs. 51.8 ± 6.0, standardized difference = 0.16) and stress levels (PSS-10: 24.3 ± 5.1 vs. 24.8 ± 4.9, standardized difference = 0.10) were well-balanced between groups.

**Table 1:** Baseline Demographic and Clinical Characteristics

Variable	VR Group (*n* = 72)	Control Group (*n* = 73)	Standardized Difference
Age (years)	36.1 ± 11.3	37.1 ± 12.5	0.08
Gender (Male)	41 (56.9%)	46 (63.0%)	0.12
Education (Secondary)	34 (47.2%)	39 (53.4%)	0.12
ASA Physical Status I	52 (72.2%)	54 (74.0%)	0.04
Surgery Duration (min)	65.4 ± 18.2	63.8 ± 17.5	0.09
Baseline Anxiety (STAI)	52.8 ± 6.6	51.8 ± 6.0	0.16
Baseline Stress (PSS-10)	24.3 ± 5.1	24.8 ± 4.9	0.10
Hypertension	8 (11.1%)	9 (12.3%)	0.04
Diabetes Mellitus	4 (5.6%)	6 (8.2%)	0.11

Data presented as mean ± SD or n (%). Standardized differences < 0.20 indicate good balance

### Primary outcome

#### Intraoperative anxiety (Primary endpoint)

The VR group demonstrated significantly lower anxiety at 30 min post-induction compared to controls (37.2 ± 15.3 vs. 52.9 ± 15.7, *P* < 0.001, Cohen's d = 1.01, 95% CI: 0.67–1.34), representing a large effect size (Table 2).

**Table 2:** Anxiety Scores (STAI) Across Time Points

Time Point	VR Group	Control Group	Mean Difference (95% CI)	*P* Value	Cohen’s d (95% CI)
Preoperative	52.8 ± 6.6	51.8 ± 6.0	1.0 (−1.4 to 3.4)	0.31	0.16 (−0.17 to 0.49)
**Intraoperative**	**37.2 ± 15.3**	**52.9 ± 15.7**	**−15.7 (−20.8 to −10.6)**	** < 0.001**	**1.01 (0.67–1.34)**
Postoperative	40.1 ± 13.5	53.3 ± 15.0	−13.2 (−17.9 to −8.5)	< 0.001	0.94 (0.60–1.27)

Primary endpoint shown in bold. Data presented as mean ± SD

### Secondary outcomes

Stress and Satisfaction (Table 3).

**Table 3:** Secondary outcomes—stress and satisfaction

Outcome	VR Group	Control Group	Mean Difference (95% CI)	*P* Value	Cohen’s d (95% CI)
Perceived Stress (PSS-10)	21.1 ± 5.3	25.9 ± 5.8	−4.8 (−6.5 to −3.1)	< 0.001	0.87 (0.53–1.20)
Satisfaction (VAS 0–100)	82.4 ± 12.1	61.3 ± 15.7	21.1 (16.6 to 25.6)	< 0.001	1.49 (1.13–1.85)

Hemodynamic Parameters (Table 4).

**Table 4:** Intraoperative hemodynamic parameters

Parameter	VR Group	Control Group	Mean Difference (95% CI)	*P* Value	Cohen’s d (95% CI)
Systolic BP (mmHg)	122.8 ± 8.1	123.6 ± 8.9	−0.8 (−3.5 to 1.9)	0.71	0.09 (−0.24 to 0.42)
Diastolic BP (mmHg)	70.4 ± 7.2	73.4 ± 7.8	−3.0 (−5.4 to −0.6)	0.19	0.40 (0.07–0.73)
Heart Rate (bpm)	85.5 ± 7.6	90.0 ± 7.8	−4.5 (−8.5 to −0.5)	0.028	0.58 (0.25–0.91)
SpO₂ (%)	97.4 ± 0.8	97.5 ± 1.0	−0.1 (−0.4 to 0.2)	0.50	0.11 (−0.22 to 0.44)

### Adverse events

#### Intervention adherence and fidelity

All patients in the VR group (100%) completed the full 30-min intervention without early discontinuation. VR equipment functioned properly in all cases, with consistent delivery of the standardized intervention protocol (Table 5).

**Table 5:** Intraoperative Adverse Events

Event	VR Group (n = 72)	Control Group (n = 73)	Relative Risk (95% CI)	*P* Value
Hypotension	5 (6.9%)	3 (4.1%)	1.69 (0.43–6.64)	0.472
Bradycardia	2 (2.8%)	1 (1.4%)	2.03 (0.19–21.80)	0.543
Nausea/Vomiting	4 (5.6%)	6 (8.2%)	0.68 (0.20–2.32)	0.507
VR-Related Discomfort	5 (6.9%)	1 (1.4%)	5.07 (0.61–42.20)	0.201

Values presented as n (%). VR-related discomfort was mild in all cases and did not require intervention discontinuation

## Discussion

### Principal findings

This randomized controlled trial demonstrates that immersive VR significantly reduces intraoperative anxiety in Palestinian patients undergoing urological surgery under regional anesthesia. The primary endpoint showed a large effect size (Cohen's d = 1.01) for anxiety reduction. After Bonferroni correction for multiple secondary outcomes, stress reduction and patient satisfaction remained statistically significant, while heart rate differences did not reach corrected significance levels.

### Anxiety reduction and mechanism

Our anxiety reduction findings (Cohen's d = 1.01) align with previous VR studies in Western populations (d = 0.85–1.2) [17, 20], demonstrating cross-cultural effectiveness. Urological surgeries were selected because they involve regional anesthesia with conscious patients, high baseline anxiety due to intimate procedures, and predictable durations suitable for VR intervention.

VR engages multiple sensory modalities, diverting attention from stressful stimuli through inhibitory learning mechanisms that modulate anxiety-processing brain regions, particularly the amygdala and anterior cingulate cortex [3, 12]. The immersive quality activates parasympathetic nervous system responses, reducing cortisol and promoting relaxation. This physiological mechanism is consistent with findings from meta-analyses in pediatric populations, demonstrating VR's broad applicability for anxiety reduction [21]. Furthermore, VR's efficacy in reducing anxiety during specific procedures like dental implant surgery supports its use across diverse surgical contexts [22].

### Cultural adaptations

Despite limited VR research in Arab healthcare settings, our study achieved high acceptance (98.6% completion rate) using culturally adapted content including Arabic-language meditation and Islamic principles. The cross-cultural effectiveness suggests nature-based VR environments have universal appeal, though cultural adaptations likely contributed to exceptional acceptance rates compared to Western studies. High patient satisfaction with non-pharmacological interventions has been documented in other settings, reinforcing their value in perioperative care [23].

### Secondary outcomes

After Bonferroni correction, stress reduction (4.8 points on PSS-10) and satisfaction improvement (21.1 points on VAS) remained statistically significant, representing clinically meaningful changes. Heart rate differences, while showing a moderate effect size (Cohen's d = 0.58), did not reach corrected significance (*p* = 0.028 vs. required *p* < 0.0125), though the trend suggests potential parasympathetic activation without hemodynamic compromise.

### Clinical implementation

VR offers excellent cost-effectiveness for resource-constrained settings. The Meta Quest 2 ($350) with content licensing serves approximately 200 patients annually at $2–3 per patient, compared to $50–100 for anxiolytic medications. Break-even occurs after treating 7–10 patients, with additional benefits of avoiding sedation-related complications and medication dependencies.

### Safety and tolerability

VR demonstrated excellent safety with no serious adverse events. Mild VR-related discomfort (6.9%) was transient and required no intervention discontinuation. The intervention maintained hemodynamic stability while providing anxiety reduction benefits.

### Limitations

Key limitations include: (1) single-item satisfaction measurement due to resource constraints; (2) potential social desirability bias in Palestinian cultural context; (3) spiritual content confounding, as Islamic prayer elements may independently reduce anxiety; (4) homogeneous sample limiting generalizability; (5) single-center design; (6) retrospective registration affecting transparency; (7) inability to achieve complete patient blinding due to intervention nature.

### Clinical implications

The findings support VR integration into perioperative protocols for regional anesthesia procedures. The technology requires minimal staff training, scales easily to existing workflows, and provides non-pharmacological anxiety management particularly valuable in resource-limited settings where medication access may be restricted.

### Future research

Future studies should investigate VR effects across diverse surgical specialties and age groups, compare VR with and without spiritual content to isolate technology effects, conduct multi-center trials for enhanced generalizability, and include long-term follow-up assessments to evaluate sustained benefits beyond immediate postoperative periods.

## Conclusions

This study demonstrates that culturally adapted VR is a safe, effective, and acceptable non-pharmacological intervention for reducing intraoperative anxiety and improving patient satisfaction in urological surgery under regional anesthesia. The intervention offers significant clinical benefits with excellent cost-effectiveness for resource-constrained settings. While limitations exist, the findings support VR integration into perioperative care protocols, particularly for conscious sedation procedures.

## Supplementary Information

Supplementary Material 1.

## Data Availability

Due to ethical restrictions, datasets are not publicly available. De-identified data may be available upon reasonable request to the Scientific Research Ethics Committee, Faculty of Nursing, Cairo University (nursing@cu.edu.eg, 23,645,336–23,657,190, Reference: RHDIRB2019041701) or corresponding author, subject to committee approval.
