# Supplementary material for: Virtual reality for reducing intraoperative anxiety and improving patient satisfaction under regional anesthesia among palestinian patients: a randomized controlled trial
Source: BMC Anesthesiol. 2025 Nov 27;25:593. doi: 10.1186/s12871-025-03475-3 (PMC12659134; doi:10.1186/s12871-025-03475-3)
Supplement: Supplementary file 1 — Supplementary Material 1. [file 12871_2025_3475_MOESM1_ESM.pdf]

CONSORT 2025 Checklist (Virtual Reality for Reducing Intraoperative Anxiety and Improving Patient Satisfaction Under Regional Anesthesia Among Palestinian Patients: A Randomized Controlled Trial)

Section / Topic	No	Item Description	Reported in Manuscript
**Title and Abstract**	1a	Identification as a randomized trial	Title, Abstract
	1b	Structured abstract (design, methods, results, conclusions)	Abstract
**Open Science**	2	Trial registration (Registry name, ID, date)	Methods 2.1
**Protocol and statistical analysis plan**	3	Where the trial protocol and statistical analysis plan can be accessed	Declarations section (ClinicalTrials.gov link provided)
**Data sharing**	4	Data sharing: where/how de-identified data, code, and materials can be accessed	Declarations section
**Funding and conflicts of interest**	5a	Sources of funding and role of funders in study design, conduct, analysis, reporting	Declarations (Funding)
	5b	Financial and other conflicts of interest of the authors	Declarations (Competing interests)
**Introduction**	6	Scientific background and rationale	Introduction
**Objectives**	7	Specific objectives related to benefits and harms	Introduction
**Methods**	8	Details of patient or public involvement in the design, conduct, and reporting	Methods 2.11

**Trial Design**	9	Description of trial design (e.g., parallel, crossover), allocation ratio, framework (superiority/equivalence/non-inferiority)	Methods 2.1
**Protocol Changes**	10	Important changes to the trial after commencement, including non-prespecified outcomes or analyses with reasons	Methods 2.12
**Trial setting**	11	Settings (e.g., community, hospital) and locations (e.g., countries, sites) where the trial was conducted	(Methods 2.1)
**Eligibility criteria**	12a	Eligibility criteria for participants	Methods 2.2
	12b	If applicable, eligibility criteria for sites and for individuals delivering the interventions (e.g., surgeons, physiotherapists)	Methods 2.3, 2.6
**Intervention and comparator**	13	Intervention and comparator with sufficient details to allow replication. If relevant, where additional materials can be accessed	Methods 2.6–2.7
**Outcomes**	14	Prespecified primary and secondary outcomes, including measurement variables, analysis metrics,<br>method of aggregation, and time points	Methods 2.9
**Harms**	15	How harms were defined and assessed (e.g., systematically, non-systematically)	Methods 2.9
**Sample size**	16a	How sample size was determined, including all assumptions supporting the calculation	Methods 2.8

	16b	Explanation of any interim analyses and stopping guidelines	Methods 2.8
**Randomisation**	17a	Who generated the random allocation sequence and the method used	Methods 2.3
	17b	Type of randomisation and details of any restriction (e.g., stratification, blocking and block size)	Methods 2.3
**Allocation concealment**	18	Mechanism used to implement the random allocation sequence, including steps to conceal it until<br>assignment	Methods 2.3
**Implementation**	19	Whether personnel who enrolled participants and those assigning them to interventions had access to the sequence	Methods 2.3
**Blinding**	20a	Who was blinded after assignment to interventions (e.g., participants, care providers, outcome assessors,<br>data analysts)	Methods 2.1, 2.3
	20b	If blinded, how blinding was achieved and description of similarity of interventions	Methods 2.3
**Statistical methods**	21a	Statistical methods used to compare groups for primary and secondary outcomes, including harms	Methods 2.10
	21b	Definition of who is included in each analysis (e.g., all randomized participants), and in which group	Results 3.1 + Flow diagram
	21c	How missing data were handled in the analysis	Methods 2.10
	21d	Methods for any additional analyses (e.g., subgroup or<br>sensitivity analyses), distinguishing prespecified from post hoc	Methods 2.10

**Participant flow**	22a	For each group, the numbers randomized, receiving intended intervention, and analyzed for the primary outcome	Results 3.1 + Flow diagram
	22b	For each group, losses and exclusions after randomization, with reasons	Results 3.1 + Flow diagram
**Recruitment**	23a	Dates defining recruitment and follow-up periods	Methods 2.1 (June–Sept 2024)
	23b	Why the trial ended or was stopped, if relevant	Methods 2.13
**Intervention & comparator**	24a	Intervention and comparator as actually delivered (including fidelity, adherence, who delivered)	Methods 2.6–2.7
	24b	Concomitant care received by each group during the trial	Methods 2.5, 2.7
**Baseline data**	25	Table showing baseline demographic and clinical characteristics for each group	Table 1 (corrected numbering)
**Numbers analysed, outcomes and estimation**	26	For each primary and secondary outcome, by group: ● number of participants included in the analysis ● number of participants with available data at the outcome time point ● result for each group ● estimated effect size and its precision (e.g., 95% CI) ● for binary outcomes, presentation of both absolute and relative effect size	Tables 2, 3, 4, 5 (corrected numbering)
**Harms**	27	All harms or unintended events in each group	Table 1 (Results 3.7)
**Ancillary analyses**	28	Any other analyses performed, including subgroup and sensitivity analyses, distinguishing prespecified from post hoc	Methods 2.10

**Discussion – Interpretation**	29	Interpretation consistent with results, balancing benefits and harms, and considering other relevant evidence	Discussion 4.1–4.4
**Discussion –<br>Limitations**	30	Trial limitations, addressing sources of potential bias, imprecision, generalisability, and, if relevant, multiplicity of analyses	Discussion 4.7
